# Assessing the Role of Social Bots During the COVID-19 Pandemic: Infodemic, Disagreement, and Criticism

**DOI:** 10.2196/36085

**Published:** 2022-08-25

**Authors:** Victor Suarez-Lledo, Javier Alvarez-Galvez

**Affiliations:** 1 Department of Biomedicine, Biotechnology and Public Health University of Cadiz Cádiz Spain; 2 Computational Social Science DataLab, University Research Institute on Social Sciences University of Cadiz Jerez de la Frontera Spain

**Keywords:** infodemics, social media, misinformation, epidemics, outbreaks, COVID-19, infodemiology, health promotion, pandemic, chatbot, social media bot, Twitter stream, Botometer, peer support

## Abstract

**Background:**

Social media has changed the way we live and communicate, as well as offering unprecedented opportunities to improve many aspects of our lives, including health promotion and disease prevention. However, there is also a darker side to social media that is not always as evident as its possible benefits. In fact, social media has also opened the door to new social and health risks that are linked to health misinformation.

**Objective:**

This study aimed to study the role of social media bots during the COVID-19 outbreak.

**Methods:**

The Twitter streaming API was used to collect tweets regarding COVID-19 during the early stages of the outbreak. The Botometer tool was then used to obtain the likelihood of whether each account is a bot or not. Bot classification and topic-modeling techniques were used to interpret the Twitter conversation. Finally, the sentiment associated with the tweets was compared depending on the source of the tweet.

**Results:**

Regarding the conversation topics, there were notable differences between the different accounts. The content of nonbot accounts was associated with the evolution of the pandemic, support, and advice. On the other hand, in the case of self-declared bots, the content consisted mainly of news, such as the existence of diagnostic tests, the evolution of the pandemic, and scientific findings. Finally, in the case of bots, the content was mostly political. Above all, there was a general overriding tone of criticism and disagreement. In relation to the sentiment analysis, the main differences were associated with the tone of the conversation. In the case of self-declared bots, this tended to be neutral, whereas the conversation of normal users scored positively. In contrast, bots tended to score negatively.

**Conclusions:**

By classifying the accounts according to their likelihood of being bots and performing topic modeling, we were able to segment the Twitter conversation regarding COVID-19. Bot accounts tended to criticize the measures imposed to curb the pandemic, express disagreement with politicians, or question the veracity of the information shared on social media.

## Introduction

Social media has radically changed the way we live and communicate. These new communication platforms offer unprecedented opportunities to improve many aspects of our lives, including public health [1,2]. They are useful to improving our access to evidence-based health information that can be fundamental to promoting healthy habits and fostering risk prevention [2]. In addition, the progressive growth of web-based, health-related knowledge and content has been found to be useful for patients who need to acquire medical skills and enhance their self-efficacy for adherence to treatments or therapies as well as for disease prevention [3].

Nevertheless, social media has also opened the door to new social and health risks [4,5]. Policies to mitigate misinformation and false health rumors are becoming increasingly common. In fact, some of the most widespread social media platforms such as Facebook, Instagram, and Twitter have implemented policies to combat the spread of misinformation regarding the COVID-19 pandemic. However, the web-based ecosystem is still overrun with health myths, hoaxes, and fake news stories that—either consciously or unconsciously—are propagated by social media users for different purposes. These messages can lead to attitude and behavior changes that may result in inadequate health decisions [6,7]. The effect of health misinformation has also been found to be determinant in health decision-making during risky situations and outbreaks such as the H5N1, Ebola, and Zika [5] viruses and the more recent COVID-19 pandemic [8,9]. Misleading messages have even hampered public health actions taken to tackle outbreaks [10-12]. For instance, in the context of the COVID-19 pandemic, misleading information has been detected regarding the origin of the virus, the potential treatments and protective measures available, and the real impact of the disease [13]. In one sample of tweets relating to COVID-19, 24.8% of the tweets included misinformation and 17.4% included unverifiable information [13]. Recently, much of the misinformation during the pandemic has focused on the debate regarding the vaccination process and the subsequent doubts the new vaccines have raised among the population [14].

Therefore, the role of social media during the COVID-19 pandemic has been critical. Although these new platforms have been useful to keep the public informed during the most critical moments of the pandemic, the responses by health authorities to combat the outbreak have been followed by a massive “infodemic,” recently defined as “an overabundance of information—some accurate and some not—that makes it hard for people to find trustworthy sources and reliable guidance when they need it” [15]. Information consumption, opinion formation, and social contagion processes relating to COVID-19 across the social media ecosystem have become a major challenge for researchers [16], since these processes can strongly affect people’s behavior and reduce the effectiveness of the countermeasures implemented by governments and health organizations [17].

Recently, misinformation dynamics have increased their complexity due to the emergence of so-called “social bots” (ie, automated web-based accounts). The role of social bots in the spread of misinformation on social media platforms has been widely recognized during political campaigns and election periods [18] and in relation to health debates, especially during health crises [19]. Regarding health communication on social media platforms, some studies have found that social bots are used to promote certain products to increase company profits and favor certain ideological positions [20] or contradict health evidence [21,22]. Bots have certain behavioral characteristics that make them potential super-spreaders of misinformation (eg, excessive posting and frequent retweeting of emerging news and tagging and mentions of influential topics or relevant figures) [20,23,24]. These accounts often use amplification as a strategy for the dissemination of content that misinforms based on the interests of the creators of these automatic accounts [25], although they are also often used as a tool to generate disagreement and social polarization [22].

The activity of social bots has dramatically increased in the context of the COVID-19 infodemic [25] due to their participation in the debate on the health measures to control the pandemic and the vaccines that have emerged during this period [26]. To date, it has been established that the progressive proliferation of social bots (and particularly unverified accounts) in the complex social media ecosystem may contribute to the increased spread of COVID-19 misinformation and the subsequent evolution of the pandemic, either by amplifying messages of dubious quality or generating polarization in relation to controversial issues [25]. However, a better understanding is needed on the role of these bots in the COVID-19 infodemic [27]. In an attempt to fill this knowledge gap, this study aimed to explore the role of social bots during the early stages of the COVID-19 pandemic. Our objective was to answer 3 basic questions: (1) What were the main conversation topics during the outbreak of COVID-19 on Twitter? (2) How do these topics vary depending on the information source (nonbots, bots, or self-declared bots)? and (3) How does the general tone of the conversation vary depending on the source?

## Methods

### Data Collection

Data collection started on March 16 and ended on June 15, 2020, using the Twitter streaming API with the following hashtags: *covid_19*, *covid19*, *covid*, and *coronavirus*. These hashtags were used during this period to capture the conversation during the first wave of COVID-19. To simplify the subsequent analysis, only tweets written in the English language were selected. The resulting data sample contained approximately 14 million tweets from about 285,000 different Twitter accounts.

### Bot Classification

We used *Botometer* (formerly *BotOrNot*; OSoMe project) [28] to obtain the likelihood of whether each account is a bot or not. *Botometer* is a publicly available service that leverages more than 1000 features to evaluate the extent to which a Twitter account exhibits similarity to the known characteristics of social bots. As in other studies [29,30], 0.8 is the score used to classify an account as a bot. In addition, the percentage of bot accounts in benchmark studies is between 9% to 15% of the total number of accounts on Twitter [31]. In our case, this score classified approximately 14% of the accounts as bots.

In addition to the overall likelihood of being a bot, *Botometer* also gives specific scores for 6 different bot types: echo chamber, fake follower, financial, self-declared, spammer, and other. Given the differing nature of social bots, it was considered necessary to draw a distinction between self-declared bots and other types of bots. Self-declared bots are extracted from Botwiki [28,32].

### Topic Modeling

Finally, together with the bot classification, we also applied topic-modeling techniques. This unsupervised classification approach allows the classification of texts, using techniques such as clustering to find groups of texts with similar content. In this case, we used latent dirichlet allocation (LDA), a popular topic-modeling technique which considers each document as a random mixture of various topics and each topic as a mixture of words [33].

To correctly interpret the results, we considered the distribution of the topics within the corpus, the keywords of each of the topics, and the intertopic distance [34]. Based on this, the most common topics of the different documents in the corpus were extracted. For each topic, we obtained the most relevant words and the 50 most characteristic tweets according to the model. We then carried out an inductive qualitative process to characterize each topic, followed by a descriptive process to codify the information [35]. Discrepancies were shared and resolved by mutual agreement. We also analyzed the distribution of the different types of accounts in the topics. This approach allowed us to determine the main conversation topics [36] and the most common ones for each type of account.

In addition, we plotted an intertopic distance map [34] to visualize the topics in a 2D space. The area of the topic circles is proportional to the number of tokens (ie, single words) that belong to each topic across the dictionary. The circles were plotted using a multidimensional scaling algorithm based on the words they comprise, with the topics that are closer together having more words in common.

### Sentiment Analysis

For each of the groups, we used sentiment analysis to examine the tone or sentiment associated with the content. Sentiment analysis is an area of knowledge in the field of natural language processing, text analysis, and computational linguistics used to identify and extract subjective information from resources. In the case of text mining, sentiment analysis involves automatically mass-classifying documents based on the positive or negative connotation of the language in the document [37].

For the sentiment extraction, we used Valence Aware Dictionary and Sentiment Reasoner (VADER), a rule-based tool specifically attuned to sentiments expressed on social media platforms [38]. VADER uses a combination of sentiments associated with lexicons that are generally labeled according to their semantic orientation as positive or negative. Unlike other text analysis tools, VADER works well on texts extracted from social media platforms, because it does not need as much text as other tools [39-41].

Another feature of this method is the output value. Most sentiment analyses classify texts as positive, negative, and neutral; for example, texts with a predominance of words, expressions, or ways of writing perceived as positive are classified as positive. However, the method used here returns a sentiment score between –1 and 1, allowing a higher level of comparison between the different types of accounts.

## Results

### Bot Classification

Table 1 shows the resulting classification. If the probability of an account being a bot is lower than 0.8, we considered it as a normal user (ie, nonbot). If the probability of an account being a self-declared bot is higher of 0.8, we classified it as a self-declared bot. Accounts with the probability of being a bot higher than 0.8 and the probability of being a self-declared bot lower than 0.8 were classified as bots. Of the 205,298 accounts, most (n=187,992, 91.6%) were normal users; 4.2% (n=8616) were classified with a high likelihood of being bot accounts; and 4.2% (n=8690) were classified as self-declared bots. Bot accounts posted an average of 123.3 tweets per user. During the 3-month time window, accounts classified as self-declared bots posted a slightly lower average of 121.1 tweets per user. However, accounts classified as having a low likelihood of being bots posted 42.5 tweets per user. These differences between the mean values were statistically significant (*F*_2,284,814_=1056; *P*<.001). As also noted in Broniatowski et al [22], the most active accounts on average were those classified as bots.

**Figure 1:**
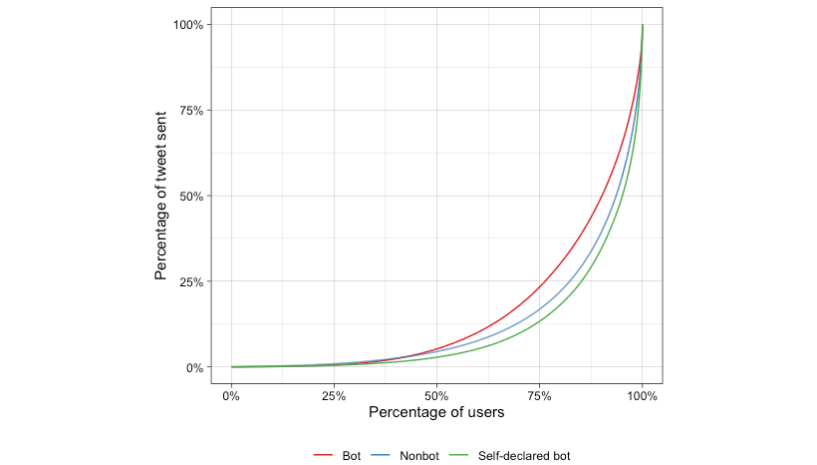
Lorenz curve showing inequality in the number of tweets.

Not all groups contributed to the same extent. Likewise, the contribution of the participants in the global discussion was highly unequal. The Gini index was used to measure this inequality. This index is a measure of the distribution, with a higher Gini index indicating greater inequality. Figure 1 shows these distributions, with a Gini index of 0.786 for self-declared bots, 0.744 for nonbots, and 0.686 for bots. Self-declared bots had the most unequal distribution: 75% (6517/8690) of self-declared bots posted 12.5% (131,559/1,052,471) of the total number of tweets. In contrast, 75% (6462/8616) of the bot accounts posted 25% (265,499/1,061,997) of the total tweets.

**Table 1:** Distribution of bot classification.

Source	Account (N=205,298), n (%)	Tweet (N=10,098,455)		
		n (%)	Mean	Median
Nonbot	187,992 (91.6)	7,983,987 (79.1)	42.5	9.0
Bot	8616 (4.2)	1,061,997 (10.5)	123.3	35.5
Self-declared bot	8690 (4.2)	1,052,471 (10.4)	121.1	15.0

In the case of self-declared bots, the most active accounts spread official data (the number of COVID-19 cases and mortality, etc). Second, several of these accounts were digital magazines or independent news agencies. The descriptions of these accounts mentioned that they created messages to provide periodic reports on the situation and communicate the global evolution of COVID-19 or substantial changes in the evolution of the pandemic. Many of these accounts indicated that their purpose was informative. Given this situation, these profiles were separated from those classified as regular bots in the analysis.

The descriptions of the accounts classified as bots were very different from each other. Many accounts identified themselves with technology companies. Others identified themselves as activists, either political, environmental, or even military. These accounts tweeted about the pandemic, the political measures taken, or complaints about the situation resulting from inaction.

### Topic Modeling

After classifying the accounts, the topics were extracted using LDA. To select the correct number of topics, we relied on the coefficient of variation, which measures the coherence between the topics inferred by a model. In other words, the coefficient indicates which combination of topics is the most coherent. Higher values indicate that the topics are semantically interpretable. Topic coherence measures score a single topic by measuring the degree of semantic similarity between high-scoring words in the topic. This concept brings together several measures to assess the coherence between the topics. To choose the number of topics, the LDA model was reapplied with different outputs, and those with the highest coefficient of variation were selected (Multimedia Appendix 1). In total, 18 topics were extracted and plotted using the intertopic distance map.

**Figure 2:**
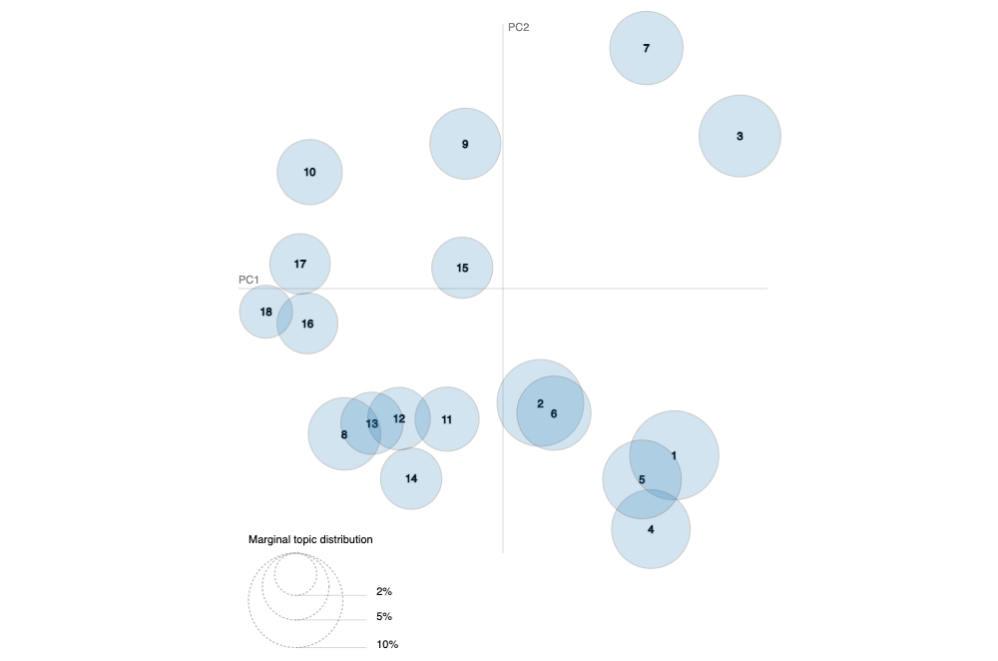
Intertopic distance map. PC: principal component.

In the intertopic distance map below (Figure 2), each bubble represents a topic. Each topic was assigned a number depending on the number of tweets inside it. Accordingly, Topic 1 had a higher percentage of tokens than Topic 2 and so on. The larger the bubble, the higher the number of tokens classified in this topic. The further the topics are away from each other, the more different they are. Therefore, there are not many differences between 2 nearby topics. On the contrary, there are greater differences if they are further apart.

**Figure 3:**
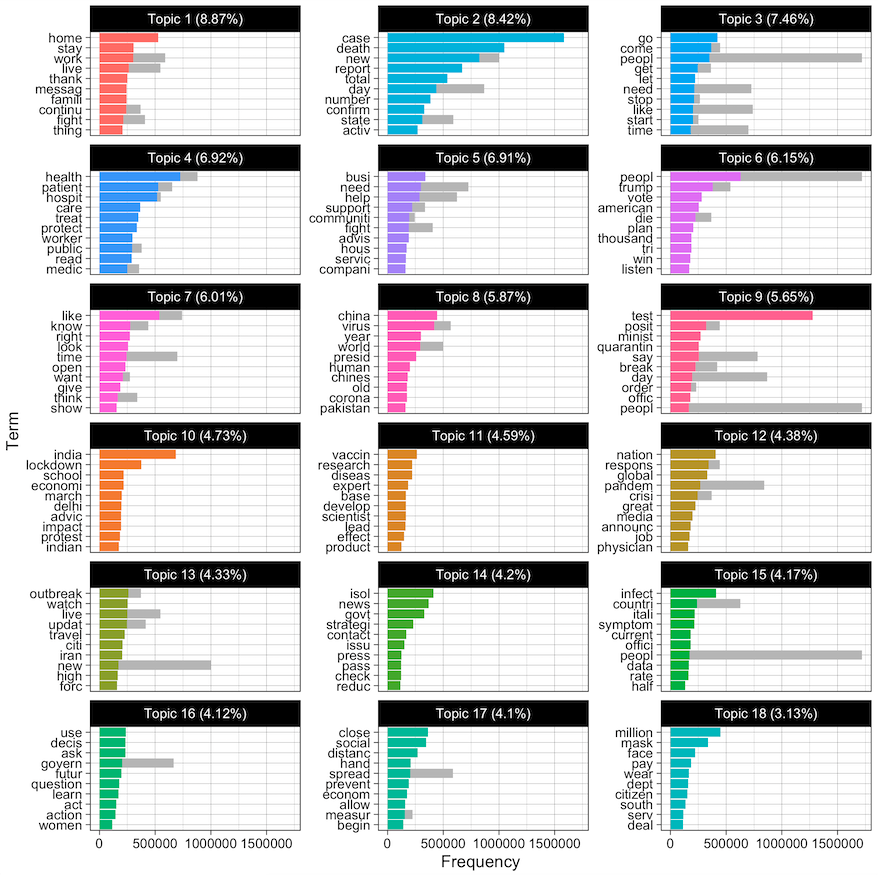
Word distribution along with topics.

We also plotted the most common terms in a bar chart (Figure 3). The terms were sorted according to the number of times they appear. The colored bars show the estimated number of times a term is in each topic. The grey bars represent the overall frequency of each term in the corpus. When interpreting the results, it is not only necessary to consider the most common terms but also the most salient terms. Saliency is the product of weighting the probability of a word, *P*(w), by its distinctiveness, a measure of how informative the specific term is to determine the generative topic. Saliency is therefore a measure of the degree to which the word appears a small number of times or not at all in other topics [36].

We then carried out the qualitative phase, with both authors agreeing in 89% (16/18) of the cases. Table 2 shows the results obtained in the classification.

**Figure 4:**
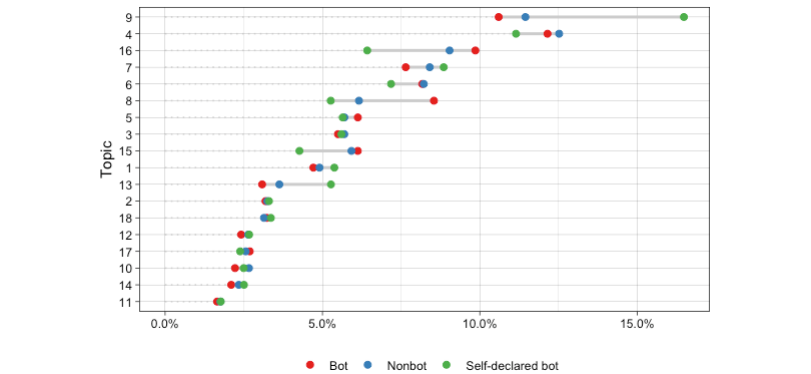
Account distribution within topics.

In Figure 4, each line is a topic, and each dot represents the percentage of accounts in each topic. Topic 1 contained tweets with information on the outbreak. The messages were focused on providing information about the advance of the pandemic and what actions need to be taken to stop it. The most common words were *stay*, *home*, and *family.* Other tweets shared this kind of information but for specific regions. For example, Topic 9 was focused on regions in Africa, and Topic 13 was focused on the lockdown in India.

These 2 topics have the most substantial differences between self-declared bots and the rest of the accounts. Topic 9 accumulated the highest percentage (1581/8690, 18.2%) of self-declared bot accounts, compared to bots (896/8616, 10.4%) and nonbots (20,115/187,992, 10.7%). Likewise, the percentage of self-declared bots in Topic 13 is 5.7% (495/8690), whereas the percentage for bots is 3.4% (293/8616) and 3.8% (7144/187,992) for nonbots.

Topic 2 contained information about the evolution of the pandemic. This topic was focused on the second wave and information on the number of deaths. The most common keywords were *case*, *death*, *report*, and *total*. In the following topics, the model groups’ contents were related to specific measures to curb the pandemic. Topic 3 mentioned the lack of testing. Some topics reminded people to stay at home (Topic 4), of the importance of wearing a face mask (Topic 11), or of washing one’s hands (Topic 12).

Other messages were related to US politics or President Trump. Most of the tweets in Topic 17 were about decisions by the US Congress. Topic 18 mentioned certain national political scandals. Topic 8 was focused on criticizing President Trump’s policies. These tweets cast President Trump as a liar and irresponsible. Some of the most common keywords were *president*, *Trump*, *China*, *virus*, *year*, and *world*. This topic had the biggest difference between the percentage of bots and the rest of the accounts. In Topic 16, most of the tweets mentioned the lack of honesty of the US President. There were also complaints about the need to share true information and disregard rumors (Topic 15). In these last 2 topics, the percentage of bot accounts was slightly higher than the rest of the accounts.

**Table 2:** Main idea for each topic.

ID	Topic
1	News about coronavirus
2	Second wave and vaccines
3	Complaints about lack of COVID-19 testing
4	Stay home
5	China and its relationship with the virus
6	Respect health care workers
7	Financial aid and charity during the pandemic
8	Trump and the pandemic
9	Reporting positive cases in Maharashtra and Africa
10	Pointing out that COVID-19 is different from influenza
11	Wearing face masks
12	Tips to prevent spreading COVID-19
13	Lockdown in India
14	Death of a famous person
15	Calls for real leadership
16	A call for honesty
17	Decisions in the US Congress
18	A national scandal

### Sentiment Analysis

**Figure 5:**
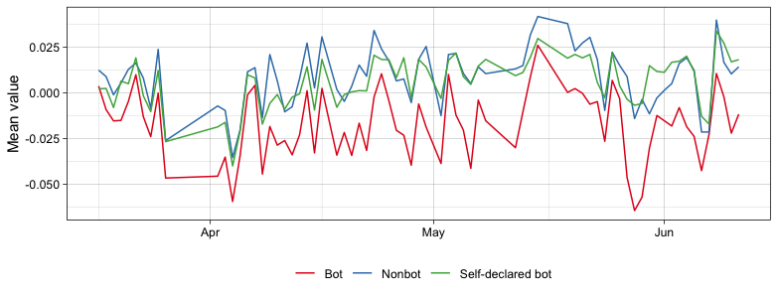
Mean value for Valence Aware Dictionary and Sentiment Reasoner (VADER) sentiment analysis.

The mean value of the VADER score for each group was 0.0109 (SD 0.414) for nonbots, 0.00784 (SD 0.383) for self-declared bots, and –0.0155 (SD 0.427) for bots. An ANOVA test was used to check for statistically significant differences in the mean values of the groups (*F*_2,284,814_=5216; *P*<.001). Figure 5 shows the evolution of the average scores over the period. The mean value was almost always lower in the case of bots, indicating a greater presence of words associated with negative feelings in this group. Accounts classified as self-declared bots were closer to values of 0. On the other hand, accounts classified as bots scored negatively.

These differences in sentiment between nonbots, self-declared bots, and undeclared bots are better understood if we consider the different topics that made up these conversations. Although most of the tweets posted by nonbots were focused on sharing the situation people were experiencing due to the outbreak, self-declared bots tended to inform and post news on the outbreak all over the world, and undeclared bots were generally focused on criticizing political measures, interpersonal blame between senators or governors, and criticism directed at governments or political leaders in relation to the mismanagement of the health crisis. At this point in the analysis, it seemed more likely that undeclared bots spread messages of disagreement, criticism, and complaints regarding the political and health authorities in view of the difficulties to adequately control the pandemic.

## Discussion

### Principal Findings

This study has allowed an assessment of the role of social bots on Twitter during the early stages of the COVID-19 pandemic. There were consistent differences between the different account types identified (self-declared bots, undeclared bots, and nonbots). Although the percentage of undeclared bots on Twitter is relatively low compared to the large number of human users, it has been established that bots are generally linked to web-based conversations characterized by controversy and polarization. In this sense, the role of these automatic agents is far from negligible, considering the role they play in the amplification of ideas and opinions that generate conflict in our societies [42,43].

The classification adopted has allowed the comparison of the different topics arising in the conversations of 3 different profiles of Twitter users during the initial months of the pandemic. Furthermore, to the best of our knowledge, this study has several advantages compared to other works that analyze sentiment in a general manner and regardless of the information source and type [44-46]. First, our study provides additional information on the information sources (nonbots, self-declared bots, and bots), particularly concerning the credibility of the different Twitter users. Second, it allows a deeper analysis of the Twitter conversation based on topics and the associated sentiments during the outbreak of the COVID-19 pandemic. Third, the comparison of the topics according to source shows there is internal consistency between the different types of accounts. Therefore, the differentiation of topics and sentiments linked to different Twitter user accounts (and particularly those relating to bots) is relevant for the identification, characterization, and monitoring of possible sources of disinformation that could emerge in the event of an infodemic [47].

On the other hand, the sentiment analysis also gives an idea of the strategy of undeclared bots or automated accounts in the context of the first months of the COVID-19 pandemic. Our study shows that social bots were used to criticize and harass political opponents rather than to provide useful information on health measures and self-protection behavior in a context where quality information was sorely needed in the face of widespread misinformation [47]. In-line with our results, a recent study indicates that right-wing self-media accounts and conspiracy theorists may give rise to this opinion polarization, whereas malicious bots may foster the diffusion of noncredible information [42]. We have not found large amounts of misinformation on health issues but rather major divisions regarding political decision-making processes and the measures to address the COVID-19 pandemic (eg, vaccines and protective measures, etc). In this sense, the conversation on automated accounts is directed more toward generating conflict and disagreement [43].

Despite these findings, additional evidence is needed to determine the social and health impacts of the misuse of social bots during the early months of the pandemic. Likewise, it is necessary to determine to what extent these agents have hindered the prevention and control of the health crisis by the different governments. In any case, this is a new working hypothesis that remains open and should be analyzed in detail in future studies.

### Limitations and Strengths

This study is subject to several limitations. First, the data collected from Twitter is limited by the technical characteristics of the Twitter streaming API. Although the streaming API is more accurate than the REST API, it never returns the total number of tweets about the conversation [48]. Moreover, due to technical limitations, it is impossible to analyze the entire conversation. In addition, by selecting only tweets written in the English language, the content of the conversations is strongly focused on topics in the United States and United Kingdom. Second, the period analyzed is in the early stages of the outbreak, and the conversations tended to evolve just as the pandemic did. Third, when observing self-expression over the internet, only the thoughts and feelings the users chose to express at the time can be captured, which may be strategically composed to project a public persona [49]. Still, many mental health studies have shown that social media is a valuable outlet and source of support for its users [50]. Topic modeling is a good technique to obtain a general idea of the different topics within a conversation. However, the downside of this technique is that the number of topics must be preselected. In our case, we used the coefficient of variation to identify the optimal number for each group.

On the other hand, this study also has several strengths. First, it takes into account the credibility of the information source. This aspect is rarely addressed in studies of social media platforms [2]. Second, this study analyzes conversations regarding the outbreak of a pandemic, and social media sites are hot spots in such situations [5], with users increasing their information searches on these platforms.

### Conclusions

By classifying the accounts according to the likelihood of being bots and applying topic modeling, we were able to segment the Twitter conversations regarding the COVID-19 pandemic. Nonbot accounts, for example, tended to share information or give advice on how to deal with the pandemic. The accounts declared as bots mostly shared information and statistics on the pandemic. Finally, accounts not declared as bots tended to criticize the measures imposed to curb the pandemic, express disagreement with politicians, or question the veracity of the information shared on social media platforms. We also used sentiment analysis to compare the tone of the conversations in these different groups. Self-declared bots had conversations with a neutral tone. The tone of messages written by nonbot accounts tended to be more positive than the former. On the contrary, the tone of undeclared bots was always more negative than the tone of self-declared bots. Therefore, it is necessary to work on the identification and monitoring of these agents in times of infodemics.

## Appendix

Multimedia Appendix 1 Score of the coefficient of variation by the number of topics.

## Abbreviations

LDA: latent dirichlet allocation
VADER: Valence Aware Dictionary and Sentiment Reasoner

## References

[1] McGloin AF, Eslami S (2015). Digital and social media opportunities for dietary behaviour change. Proc Nutr Soc.

[2] Chou WS, Oh A, Klein WMP (2018). Addressing health-related misinformation on social media. JAMA.

[3] Neter E, Brainin E (2012). eHealth literacy: extending the digital divide to the realm of health information. J Med Internet Res.

[4] Suarez-Lledo V, Alvarez-Galvez J (2021). Prevalence of health misinformation on social media: systematic review. J Med Internet Res.

[5] Alvarez-Galvez J, Suarez-Lledo V, Rojas-Garcia A (2021). Determinants of infodemics during disease outbreaks: a systematic review. Front Public Health.

[6] Cavallo DN, Chou WS, McQueen A, Ramirez A, Riley WT (2014). Cancer prevention and control interventions using social media: user-generated approaches. Cancer Epidemiol Biomarkers Prev.

[7] Naslund JA, Grande SW, Aschbrenner KA, Elwyn G (2014). Naturally occurring peer support through social media: the experiences of individuals with severe mental illness using YouTube. PLoS One.

[8] Funk S, Gilad E, Watkins C, Jansen VAA (2009). The spread of awareness and its impact on epidemic outbreaks. Proc Natl Acad Sci U S A.

[9] Betsch C (2013). The role of the Internet in eliminating infectious diseases. managing perceptions and misperceptions of vaccination. Article in German. Bundesgesundheitsblatt Gesundheitsforschung Gesundheitsschutz.

[10] Househ M (2016). Communicating Ebola through social media and electronic news media outlets: a cross-sectional study. Health Informatics J.

[11] Catalan-Matamoros D, Peñafiel-Saiz Carmen (2019). How is communication of vaccines in traditional media: a systematic review. Perspect Public Health.

[12] Levy JA, Strombeck R (2002). Health benefits and risks of the Internet. J Med Syst.

[13] Kouzy R, Abi Jaoude Joseph, Kraitem A, El Alam Molly B, Karam B, Adib E, Zarka J, Traboulsi C, Akl E, Baddour K (2020). Coronavirus goes viral: quantifying the COVID-19 misinformation epidemic on Twitter. Cureus.

[14] Wilson SL, Wiysonge C (2020). Social media and vaccine hesitancy. BMJ Glob Health.

[15] (2020). EPI-WIN: WHO Information Network for Epidemics. World Health Organization.

[16] Zarocostas J (2020). How to fight an infodemic. Lancet.

[17] Cinelli M, Quattrociocchi W, Galeazzi A, Valensise CM, Brugnoli E, Schmidt AL, Zola P, Zollo F, Scala A (2020). The COVID-19 social media infodemic. Sci Rep.

[18] Caldarelli G, de Nicola R, del Vigna F, Petrocchi M, Saracco F (2020). The role of bot squads in the political propaganda on Twitter. Commun Phys.

[19] Allem J, Ferrara E (2018). Could social bots pose a threat to public health?. Am J Public Health.

[20] Bessi A, Ferrara E (2016). Social bots distort the 2016 U.S. Presidential election online discussion. First Monday.

[21] Betsch C, Brewer NT, Brocard P, Davies P, Gaissmaier W, Haase N, Leask J, Renkewitz F, Renner B, Reyna VF, Rossmann C, Sachse K, Schachinger A, Siegrist M, Stryk M (2012). Opportunities and challenges of Web 2.0 for vaccination decisions. Vaccine.

[22] Broniatowski DA, Jamison AM, Qi S, AlKulaib L, Chen T, Benton A, Quinn SC, Dredze M (2018). Weaponized health communication: Twitter bots and Russian trolls amplify the vaccine debate. Am J Public Health.

[23] Shao C, Ciampaglia GL, Varol O, Yang K, Flammini A, Menczer F (2018). The spread of low-credibility content by social bots. Nat Commun.

[24] Yuan X, Schuchard RJ, Crooks AT (2019). Examining emergent communities and social bots within the polarized online vaccination debate in Twitter. Soc Media Soc.

[25] Himelein-Wachowiak M, Giorgi S, Devoto A, Rahman M, Ungar L, Schwartz H, Epstein D, Leggio L, Curtis B (2021). Bots and misinformation spread on social media: implications for COVID-19. J Med Internet Res.

[26] Zhang M, Qi X, Chen Z, Liu J (2022). Social bots' involvement in the COVID-19 vaccine discussions on Twitter. Int J Environ Res Public Health.

[27] Gallotti R, Valle F, Castaldo N, Sacco P, De Domenico M (2020). Assessing the risks of 'infodemics' in response to COVID-19 epidemics. Nat Hum Behav.

[28] Sayyadiharikandeh M, Varol O, Yang K, Flammini A, Menczer F (2020). Detection of novel social bots by ensembles of specialized classifiers.

[29] Luceri L, Badawy A, Deb A, Ferrara E (2019). Red bots do it better: comparative analysis of social bot partisan behavior.

[30] Yang K, Varol O, Davis CA, Ferrara E, Flammini A, Menczer F (2019). Arming the public with artificial intelligence to counter social bots. Human Behav and Emerg Tech.

[31] Varol O, Ferrara E, Davis C, Menczer F, Flammini A (2017). Online human-bot interactions: detection, estimation, and characterization. https://ojs.aaai.org/index.php/ICWSM/article/view/14871.

[32] Botwiki.

[33] Rehurek R, Sojka P (2011). Gensim--python framework for vector space modelling. https://www.fi.muni.cz/usr/sojka/posters/rehurek-sojka-scipy2011.pdf.

[34] Sievert C, Shirley K (2014). LDAvis: a method for visualizing and interpreting topics.

[35] Colorafi KJ, Evans B (2016). Qualitative descriptive methods in health science research. HERD.

[36] Chuang J, Manning C, Heer J (2012). Termite: visualization techniques for assessing textual topic models.

[37] Liu B (2012). Sentiment analysis and opinion mining. Synthesis Lectures on Human Language Technologies.

[38] Hutto C, Gilbert E (2014). VADER: A parsimonious rule-based model for sentiment analysis of social media text. https://ojs.aaai.org/index.php/ICWSM/article/view/14550.

[39] Elbagir S, Yang J (2019). Twitter sentiment analysis using natural language toolkit and VADER sentiment analyzer. Lecture Notes in Engineering and Computer Science.

[40] Pano T, Kashef R (2020). A complete VADER-based sentiment analysis of Bitcoin (BTC) tweets during the era of COVID-19. Big Data Cogn Comput.

[41] Adarsh R, Patil A, Rayar S, Veena K (2019). Comparison of VADER and LSTM for sentiment analysis. Int J Recent Technology Engineering.

[42] Xu W, Sasahara K (2022). Characterizing the roles of bots on Twitter during the COVID-19 infodemic. J Comput Soc Sci.

[43] Uyheng J, Carley KM (2020). Bots and online hate during the COVID-19 pandemic: case studies in the United States and the Philippines. J Comput Soc Sci.

[44] Pokharel BP (2020). Twitter sentiment analysis during COVID-19 outbreak in Nepal. SSRN Journal.

[45] Rosenberg H, Syed S, Rezaie S (2020). The Twitter pandemic: the critical role of Twitter in the dissemination of medical information and misinformation during the COVID-19 pandemic. CJEM.

[46] Tsai MH, Wang Y (2021). Analyzing Twitter data to evaluate people's attitudes towards public health policies and events in the era of COVID-19. Int J Environ Res Public Health.

[47] Stella M, Ferrara E, de Domenico M (2018). Bots increase exposure to negative and inflammatory content in online social systems. Proc Natl Acad Sci U S A.

[48] Wang Y, Callan J, Zheng B (2015). Should we use the sample? analyzing datasets sampled from Twitter’s stream API. ACM Trans Web.

[49] Marshall PD (2015). Intercommunication and persona: the intercommunicative public self. Int J Interdisciplinary Stud Commun.

[50] McCloud RF, Kohler RE, Viswanath K (2017). Cancer risk-promoting information: the communication environment of young adults. Am J Prev Med.

