# Supplementary material for: Assessing the Role of Social Bots During the COVID-19 Pandemic: Infodemic, Disagreement, and Criticism
Source: J Med Internet Res. 2022 Aug 25;24(8):e36085. doi: 10.2196/36085 (PMC9407159; doi:10.2196/36085)
Supplement: Multimedia Appendix 1 [file jmir_v24i8e36085_app1.pdf]

Multimedia Appendix I

From the same preprocessed data, we generated models of K topics. Figure 1 contains the score of the Coefficient of Variation by the number of topics. As can be seen in the figure, the highest value of the coefficient corresponds to the value of k = 18.

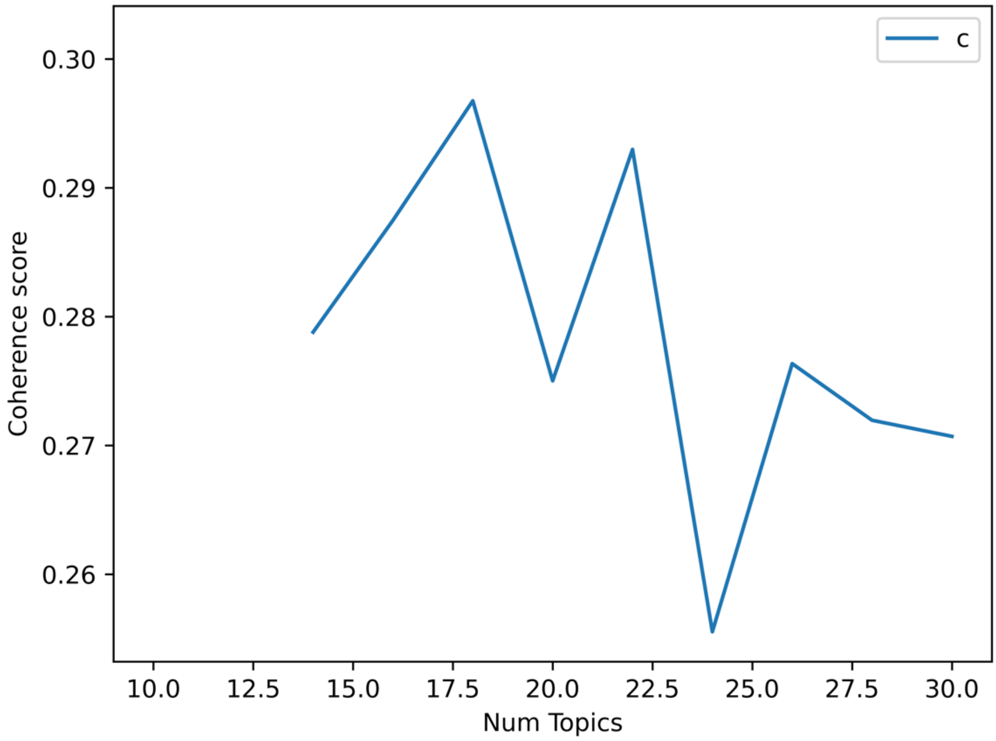

Figure 1 Coefficient of Variation
